# Reconstruction of the Temporal Correlation Network of All-Cause Mortality Fluctuation across Italian Regions: The Importance of Temperature and Among-Nodes Flux

**DOI:** 10.3390/e25010021

**Published:** 2022-12-23

**Authors:** Guido Gigante, Alessandro Giuliani

**Affiliations:** 1 Radiation Protection and Computational Physics, Istituto Superiore di Sanità, 00161 Rome, Italy; 2 Environment and Health Department, Istituto Superiore di Sanità, 00161 Rome, Italy

**Keywords:** complex networks, dynamical systems, epidemiology, time series

## Abstract

All-cause mortality is a very coarse grain, albeit very reliable, index to check the health implications of lifestyle determinants, systemic threats and socio-demographic factors. In this work, we adopt a statistical-mechanics approach to the analysis of temporal fluctuations of all-cause mortality, focusing on the correlation structure of this index across different regions of Italy. The correlation network among the 20 Italian regions was reconstructed using temperature oscillations and traveller flux (as a function of distance and region’s attractiveness, based on GDP), allowing for a separation between infective and non-infective death causes. The proposed approach allows monitoring of emerging systemic threats in terms of anomalies of correlation network structure.

## 1. Introduction

The monthly-based all-cause death rate fluctuations of the 20 Italian regions are highly correlated in time. This happens even in the absence of recognisable macroscopic parameters, such as massive epidemics. In this work, we tried and built a phenomenological model of the observed between-region correlations based on the traveller flux among the network having, as nodes, the regions and, as edges, the mutual traveller fluxes estimated by a simple exponential model having the distance between regions and Gross Domestic Product (GDP) as major determinants. The above model was complemented by the well-known biphasic effect of temperature on all-cause mortality [1,2,3,4,5]. The problem can be interpreted as the reconstruction of a network wiring in which the between nodes (regions) edge strength corresponds to the observed temporal correlation of the relative death rate fluctuations in time (*Y*-network) by the network wiring generated by the combination of between nodes fluxes and temperature effects (*X*-network).

The strategy of analysis was as follows: the (extremely high) between-region correlation was normalised by what was expected by the observed (well-known) biphasic effect of seasonality. The crude effect of seasonality (when partially out) had, as a consequence, the effect of lowering correlations, but we still have a very high residual correlation for a more refined model.

The biphasic effect (high mortality in winter and summer) on all-cause mortality was hypothesised as derived from an infective component prevailing in winter and a non-infective component prevailing in summer. This interpretation stems from the higher diffusion of viral infections in winter and cardiovascular (often from older people’s dehydration) in summer. The winter (infectious) component was modelled using the between regions travellers flux (exponentially decaying with distance) complemented by the ‘attractiveness’ of each region proportional to its GDP. Thus we generated a ‘between-region flux network’ using a SIR-like model. The summer (non-infectious) model was formalised using a linear function of the month-specific average temperature of each region. This allows us to take into consideration the effect of local heat waves.

A model encompassing the above-sketched elements (*X*-network) was fitted to the observed death rates, producing the correlation network (*Y*-network). This minimalistic model was able to reconstruct the death rate oscillation in time and the observed between-region correlation network with high fidelity (corr = 0.993 and corr = 0.841, respectively).

In this work, we demonstrate that weighted edge correlation networks are a very powerful method in epidemiological studies, allowing the tracing of the dynamics of mortality (morbidity) patterns and potentially discovering anomalies relevant to public health. Taking into account the most general definition of a system as ‘...a set of interacting units with relationships among them’ [6], we can safely state that Italy, as for death-rate fluctuations, due to the high temporal correlation among its regions, is a proper system. This allows for the sensible use of second-order statistics (such as correlations), adding unique information content to environmental and epidemiological studies that, in the great majority of cases, rely on the exploitation of a single variable (e.g., death rate fluctuations in a given area) in terms of a set of covariates (e.g., pollution, age structure, etc.).

## 2. Materials and Methods

In order to model the possible order parameters shaping the observed (and partially unexpected) very dense among regions correlation structure of the monthly-based 2011–2019 time series of all-cause mortality, we tried to keep to a minimum both the a priori hypotheses and the number of fitted parameters. This modelling choice was dictated by both the lack of any strong theory on all-cause mortality and to avoid overfitting problems.

Thus we limit ourselves to inserting, as ‘explanatory variables’, the two-phase effect of temperature and an index derived by commuter flux among different regions modulated by the GDP of each region (considered a proxy of region attractiveness).

In the following, we will call $n_{im}$ the number of deaths recorded in region i during the *m*-th month of recording; accordingly, we will denote $T_{im}$ as the average temperature in the same region during the same month.

### 2.1. Bi-Phasic Effect of Temperature

To account for temperature effects, we assume that $n_{im}$ is Poisson distributed with $\langle n_{im}\rangle =\lambda_{i}\left(T_{im}\right)$:

(1) $$\lambda_{i}^{\mathrm{biphasic}}\left(T\right)=e^{-a_{c,i}\;T+b_{c,i}}+e^{a_{h,i}\;T+b_{h,i}},$$

where $a_{c,i}>0$ and $a_{h,i}>0$ (*c* and *h* stand for ‘cold’ and ‘hot’, respectively). This is a convex function with a minimum at:

(2) $$T_{\min,i}=\frac{b_{c,i}-b_{h,i}+\log\left(a_{c,i}/a_{h,i}\right)}{a_{c,i}+a_{h,i}}.$$

The four parameters ($a_{c,i}$, $b_{c,i}$, $a_{h,i}$ and $b_{h,i}$) are fitted, for each region *i*, by maximising the log-likelihood (through the scipy.optimize.minimize function, with TNC method [7,8]):

(3) $$\mathrm{LL}=\sum_{m}n_{im}\;\log\left(\lambda_{i}^{\mathrm{biphasic}}\left(T_{im}\right)\right)-\lambda_{i}^{\mathrm{biphasic}}\left(T_{im}\right).$$

The temperature $T_{im}$ is computed by associating the main administrative centre of each region with the three closest weather stations for which we have temperature readings. $T_{im}$ is then taken, for each month, as the weighted average of the three stations, with the weight proportional to the inverse of the distance between each station and the main administrative centre.

### 2.2. Analysis of Commuter Flux

We denote $c_{ij}$ as the number of daily commuters from region *j* to region *i*; and $d_{ij}$ (The inter-region distance, $d_{ij}$, we used is the distance between the main administrative centres—“capoluogo”—of each region.) as the distance between the same two regions; note that $c_{ij}$, unlike $d_{ij}$, is not symmetric. We hypothesise an exponentially decaying relationship between the flux and the distance:

(4) $$c_{ij}^{\mathrm{dist}}\left(d\right)=\kappa \;\left(e^{-\frac{d}{d_{0}}}+b_{0}\right)$$

where the parameters (κ, $d_{0}$ and $b_{0}$) are fitted by maximising the log-likelihood (through the scipy.optimize.minimize function, with the TNC method; the zeros—no commuters from region *j* to region *i*—are not included in the fit):

(5) $$\mathrm{LL}=-\sum_{i,\;j|c_{ij}>0}{\left(\log\left(c_{ij}\right)-\log\left(c_{ij}^{\mathrm{dist}}\left(d_{ij}\right)\right)\right)}^{2}.$$

Here we are assuming a log-normal distribution for $c_{ij}$ around the expected value $c_{ij}^{\mathrm{dist}}$.

Defining $c_{i:}\equiv \sum_{j}c_{ij}$—the number of commuters to region *i*, and calling ${\mathrm{GDP}}_{i}$ the GDP of region *i*, we hypothesise the existence of a linear relationship:

(6) $$c_{i:}^{\mathrm{gdp}}\left(\mathrm{GDP}\right)=\kappa \;\mathrm{GDP}$$

whose slope κ is fitted by maximising the log-likelihood (through the scipy.optimize.minimize function, with the TNC method):

(7) $$\mathrm{LL}=-\sum_{i}{\left(\log\left(c_{i:}\right)-\log\left(c_{i:}^{\mathrm{gdp}}\left({\mathrm{GDP}}_{i}\right)\right)\right)}^{2}.$$

We are assuming that $c_{i:}$ follows a log-normal distribution around the expected value $c_{i:}^{\mathrm{gdp}}$. This is in contrast with the assumption of log-normality for $c_{ij}$, since the sum of log-normal variables is not itself log-normal. Yet, in many cases, this is a good approximation [9].

Finally, we performed a fit that considers the two effects together: the exponential decay with distance and the linear dependence on the GDP of the region of destination; indicating with ${\mathrm{pop}}_{j}$ the population of region *j*, we have:

(8) $$c_{ij}^{\mathrm{fit}}=\kappa \;{\mathrm{pop}}_{j}\;{\mathrm{GDP}}_{i}\;e^{-\frac{d_{ij}}{d_{0}}}.$$

As above, the fit procedure finds the best parameters κ and $d_{0}$ by maximising the log-normal log-likelihood (through the scipy.optimize.minimize function, with the TNC method; the zeros—no commuters from region *j* to region *i*—are not included in the fit).

### 2.3. Total Flux

We hypothesise that the total flux of persons $f_{ij}$ comprises, beyond the daily commuters $c_{ij}$, an ‘episodic’ component $e_{ij}$ of more irregular movements:

(9) $$f_{ij}=c_{ij}+e_{ij}.$$

Starting from the results for $c_{ij}$, we make the assumption that $e_{ij}$ is an exponentially decaying function of the distance between regions and a linear function of the GDP of the region of arrival i and of the population of the region of departure:

(10) $$e_{ij}=\kappa^{e}\;{\mathrm{pop}}_{j}\;{\mathrm{GDP}}_{i}\;e^{-d_{ij}/d_{0}^{e}}.$$

With respect to Equation (8), we expect $d_{0}^{e}>d_{0}$, since episodic travels, in contrast with frequent ones, are likely less affected by the distance to travel.

### 2.4. Sir Network Model

The full flux-temperature model includes two different effects. The first one is related to the non-infective component of mortality:

(11) $$\lambda_{im}^{\mathrm{flux}}={\mathrm{pop}}_{i}\;\left(e^{a_{h}\;T_{im}+b_{h}}+\rho_{0i}\right)+\cdots ,$$

where $\lambda_{im}^{\mathrm{flux}}$ is the model expectation for the number of deaths in region *i* at month *m*; and $\rho_{0i}$ is a baseline mortality rate for region *i*. Note that this effect is akin to the warm-season component of Equation (1), but here in the flux-temperature model, for the sake of parsimony, we lose the individualised behaviour of each region *i*, and all regions respond to high temperatures in the same way.

The second effect takes into account the infective component of mortality. We make the simplifying assumption that, in each month, a new infectious disease starts spreading; at the end of the month, a fraction μ of the people ‘recovered’ from the disease dies; the following month, the process starts afresh. The spreading of the disease follows a SIR (Susceptible, Infected, Recovered) model [10] on the flux network. Defining the two matrices:

(12) $$\begin{array}{cc} \phi_{ij} & =\left\{\begin{array}{cc} \frac{f_{ij}}{{\mathrm{pop}}_{j}} & \mathrm{for}\;i\neq j\\ 1-\frac{\sum_{k}f_{kj}}{{\mathrm{pop}}_{j}} & \mathrm{for}\;i=j \end{array}\right. \end{array}$$

(13) $$\begin{array}{cc} {\hat{\phi }}_{ij} & ={\left(\frac{\phi_{ij}}{\sum_{k}\phi_{ik}}\right)}^{⊺} \end{array}$$

the dynamics of the model reads:

(14) $$\begin{array}{cc} {\dot{S}}_{i} & =-\sum_{l}{\hat{\phi }}_{il}\;[\frac{\beta }{{\mathrm{pop}}_{l}}\;\left(\sum_{j}\phi_{lj}\;S_{j}\right)\;\left(\sum_{j}\phi_{lj}\;I_{j}\right)] \end{array}$$

(15) $$\begin{array}{cc} {\dot{I}}_{i} & =\sum_{l}{\hat{\phi }}_{il}\;[\frac{\beta }{{\mathrm{pop}}_{l}}\;\left(\sum_{j}\phi_{lj}\;S_{j}\right)\;\left(\sum_{j}\phi_{lj}\;I_{j}\right)]-\gamma \;I_{i} \end{array}$$

(16) $$\begin{array}{cc} {\dot{R}}_{i} & =\gamma \;I_{i}, \end{array}$$

where $S_{i}$, $I_{i}$ and $R_{i}$ are the number, respectively, of susceptible, infected and recovered individuals in region *i*; β measures the rate at which susceptible individuals get infected (S→I); and γ is the rate of recovery, I→R. The model, therefore, consists of 60 coupled differential equations.

The reasoning behind the model is as follows. The term $\sum_{j}\phi_{ij}\;S_{j}$ represents the number of susceptible individuals in region *i*, at a given instant in time, due to the flux from other regions (minus the flux out of region *i* itself—the diagonal elements $\phi_{ii}$); for the infected, it is $\sum_{j}\phi_{ij}\;I_{j}$. In a classical SIR model, the number of newly infected individuals dI is given by $\beta \;\frac{S\;I}{\mathrm{pop}}$; in our case:

(17) $$dI_{l}=\frac{\beta }{{\mathrm{pop}}_{l}}\;\left(\sum_{j}\phi_{lj}\;S_{j}\right)\;\left(\sum_{j}\phi_{lj}\;I_{j}\right).$$

At the end of the day, the reverse flux $f_{ji}$ (people moving back from region *i* to region *j*) will redistribute the newly infected in proportion to the fraction of susceptible individuals contributed by each region *j*; this is given by:

(18) $$dI_{i}=\sum_{l}{\hat{\phi }}_{il}dI_{l},$$

that, together with Equation (17), gives the infinitesimal increment of infected people entering Equation (15) (first term on the left).

The initial conditions are always in the form of one ‘patient zero’ in region $i_{0}$ at time t=0, so that $S^{i}\left(t=0\right)={\mathrm{pop}}_{i}$ for $i\neq i_{0}$, and $S^{i_{0}}\left(t=0\right)={\mathrm{pop}}_{i_{0}}-1$; accordingly, $I^{i}\left(t=0\right)=0$ for $i\neq i_{0}$, and $I^{i_{0}}\left(t=0\right)=1$. Since $i_{0}$ is not known, we assume it to be a random variable distributed such that:

(19) $$p\left(i_{0}=i\right)\propto {\mathrm{GDP}}_{i};$$

this amounts to assuming that the external flux to region *i* (people coming to region *i* from outside Italy) is proportional to the GDP of the region itself.

For each month, we evolve Equations (14)–(16) for 30 days (t∈[0,30]); the equations are integrated using the Euler method, with step size dt=1day. Finally, this infective component of mortality is incorporated into the model:

(20) $$\lambda_{imi_{0}}^{\mathrm{flux}}={\mathrm{pop}}_{i}\;\left(e^{a_{h}\;T_{im}+b_{h}}+\rho_{0i}\right)+\mu \;R_{im}^{i_{0}}\left(t=30\right),$$

where with $R_{im}^{i_{0}}\left(t=30\right)$ we designate the total number of recovered individuals for region *i* at the end of month *m*, when the patient zero was located in region $i_{0}$ (all months are assumed, for simplicity, to have 30 days).

To also incorporate seasonal effects in the infective dynamics, we make β a function of the temperature:

(21) $$\beta_{im}=e^{-a_{\beta }\;T_{im}+b_{\beta }},$$

with $a_{\beta }>0$; with the additional constraint that $\beta <\frac{1}{dt}$ (the condition $\beta =\frac{1}{dt}$ amounts to having all the population infected in a single dt; larger values lead, in the Euler approximation, to unphysical solutions).

Considering Equations (9) and (11) (parameters $a_{h}$, $b_{h}$ and $\rho_{0i}$), Equation (10) ($\kappa^{e}$ and $d_{0}^{e}$), Equation (20) (μ), Equation (21) ($a_{\beta }$ and $b_{\beta }$), alongside Equations (14)–(16) (γ), the model comprises 28 parameters; of which 20 (the $\rho_{0i}$) are simply used to offset the different mortality rates in different regions (due, for example, to distinct age structures). These parameters are fitted to the data by maximising the Poisson log-likelihood:

(22) $$\mathrm{LL}=\frac{1}{N_{\mathrm{counts}}}\;\sum_{i_{0}}p\left(i_{0}\right)\;\left[\sum_{i,\;m}n_{im}\;\log\left(\lambda_{imi_{0}}^{\mathrm{flux}}\right)-\lambda_{imi_{0}}^{\mathrm{flux}}\right],$$

where $N_{\mathrm{counts}}$ is the number of terms in the sum $\sum_{i,\;m}$ (if we consider $n_{\mathrm{batch}}$ different months, having 20 regions, $N_{\mathrm{counts}}=20\cdot n_{\mathrm{batch}}$), and $p(i_{0})$ is given by Equation (19).

To this likelihood, we added two prior likelihoods to constrain the parameters of the model. The first is a soft flat prior, not-null in the range [0,0.2] for the quantity $\frac{\sum_{k}f_{ki}}{{\mathrm{pop}}_{i}}$ (see Equation (9)); the log-prior becomes quadratic outside the allowed (flat probability) range; the factor in front of the quadratic term is chosen large enough to practically prevent leaving the allowed range. This constrains the fraction of the population leaving a region every day to less than 20%. The second log-prior is quadratic in $d_{0}^{e}$ (Equation (10)):

(23) $$\log\left(p(d_{0}^{e})\right)=-9.74\cdot 10^{-7}\;{\left(d_{0}^{e}\right)}^{2}+\mathrm{const},$$

to penalise very high spatial decay constants $d_{0}^{e}$ for the episodic component of the flux.

The maximisation has been carried out, in this case, through the Adam optimiser [11], with default parameters ($\beta_{1}=0.9$ and $\beta_{2}=0.99$) and a learning rate decreasing at each optimisation step according to:

(24) $$lr\left(\mathrm{step}\right)=\frac{10^{-3}}{{\left(1+\frac{\mathrm{step}}{10^{4}}\right)}^{0.75}}.$$

The training set consists of the monthly death counts for each of the 20 regions for 96 (out of 108) randomly chosen months in the period 2011–2019. We reserved 12 months (12 + 96 = 108) as test data; these months were selected to have one exemplar of each calendar month—January to December; since the dataset spans only 9 years, 3 randomly-selected years contributed two months (6 months apart, e.g., April–October) to the test data.

At each step, $n_{\mathrm{batch}}$ months (with month, we here denote one specific month in a specific year; so, in the training set, we have 96 months) are randomly selected from the training set (the same month can appear multiple times in the batch). For each month *m*, a patient zero-region $i_{0m}$ is randomly extracted with a probability given by Equation (19). The computed log-likelihood is then:

(25) $${\mathrm{LL}}_{\mathrm{batch}}=\frac{1}{20\cdot n_{\mathrm{batch}}}\;\sum_{i,\;m,\;i_{0,m}}n_{im}\;\log\left(\lambda_{imi_{0m}}^{\mathrm{flux}}\right)-\lambda_{imi_{0m}}^{\mathrm{flux}},$$

a stochastic approximation of the total log-likelihood of Equation (22).

For the first $10^{4}$ optimisation steps, $n_{\mathrm{batch}}=10$. From that step onwards, $n_{\mathrm{batch}}=100$; and, to the log-likelihood, we added a ‘regularisation’ term:

(26) $${\mathrm{LL}}_{\mathrm{corr}}=-\frac{\upsilon_{\mathrm{corr}}}{190}\;\sum_{i>j}{\left({\mathrm{corr}}_{ij}-{\mathrm{corr}}_{ij}^{0}\right)}^{2}$$

where ${\mathrm{corr}}_{ij}^{0}$ is the actual correlation between the monthly deaths of region *i* and region *j*; whereas ${\mathrm{corr}}_{ij}$ is the corresponding correlations produced by the model (on the specific batch); factor $\frac{1}{190}$ normalises the sum $\sum_{i>j}$, which comprises 190 terms. We set $\upsilon_{\mathrm{corr}}=8.76\times 10^{2}$.

We monitored the log-likelihood (Equation (22)) on the test data during the training; since it never substantially decreased (that would suggest some level of over-fitting), we interrupted the optimisation after $10^{6}$ steps, when improvement on the training set appeared extremely slow.

All the computations were performed with custom code written in Python; core functions were just-in-time compiled, and their gradient was computed, where necessary, through the Jax package (https://github.com/google/jax, accessed on 9 December 2022). The complete code, reproducing all the reported results, can be found at https://github.com/GuidoGigante/All-cause-mortality-fluctuation-across-Italian-regions, accessed on 9 December 2022.

## 3. Results

**Figure 1:**
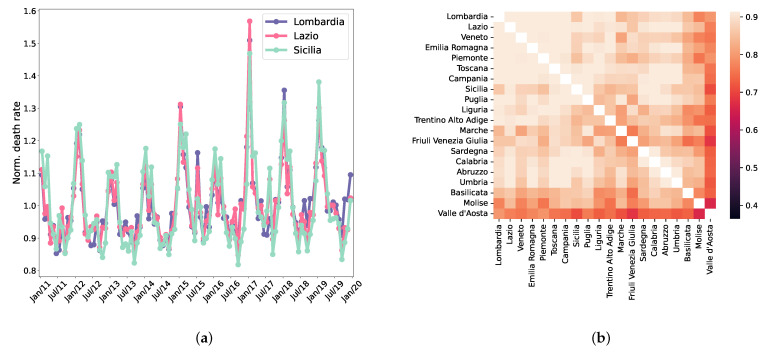
Death rates in different regions are extremely correlated. (**a**) Actual time series for three regions, normalised to have an average value equal to one. The three lines present a strikingly similar course. (**b**) Pairwise between-region correlations (regions are ordered—left to right and top to bottom—according to decreasing GDP). A trend with GDP is appreciable, with smaller and less densely populated regions (i.e., Valle d’Aosta and Molise) endowed with lower (albeit still high) correlations.

The course of monthly death rates, normalised to the mean over the entire period, is strikingly similar for different regions. This can be appreciated in Figure 1a, where we show three regions (chosen to be representative of the north—Lombardia, centre—Lazio, and south—Sicilia, of Italy).

Such observations are made more quantitative in Figure 1b, which shows the between-region correlation matrix of monthly death rate fluctuations (correlations computed on 108 data points) relative to the different regions. As evident from the figure, the between-region correlations are extremely high (0.865 ± 0.063), with smaller and less densely populated regions (i.e., Valle d’Aosta and Molise) endowed (as expected) by a lower (albeit very significant) average correlation strength (0.739 and 0.805, respectively).

To check if the bi-phasic effect of temperature was sufficient to get rid of the observed correlations (in the presence of a substantially similar age structure across the different Italian regions), a quantitative model taking into account the temperature effect was fitted to the different regions’ mortality data (see Section 2).

**Figure 2:**
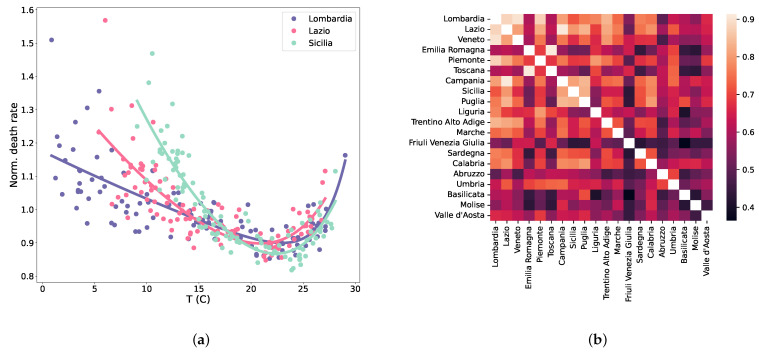
(**a**) Normalised death rates for three regions as a function of the temperature. The continuous lines are the results of a fit (see Equations (1) and (3)). (**b**) Pairwise between-region correlations for the time series of the deaths when the fitted effect of the temperature is subtracted from the raw numbers. Correlations drastically decrease (colour scale as in Figure 1b) but remain large.

All regions showed very similar relations between death rate fluctuations and temperature with the expected bi-phasic relation with two winter and summer peaks and a minimum at intermediate temperature values (spring and autumn) (see Figure 2a reporting three representative regions’ data; continuous lines: see Equation (1)).

By normalising the time series of death rate fluctuations by temperature effect, the between-region correlation drastically decreases (0.63 ± 0.12), therefore, confirming the expected effect of temperature on mortality (Figure 2b; colour scale as in Figure 1b). Notwithstanding that the residual entity correlation is still high, asking for some other relevant factor to be taken into consideration.

**Figure 3:**
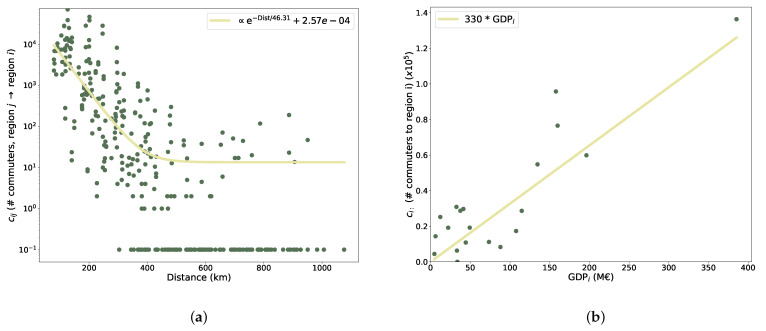
Determinants of the commuters flux. (**a**) The flux between two regions decays exponentially with the distance to travel (continuous line, exponential fit; see Equation (4)). The points at the bottom of the graph are zeros (not allowed in logarithmic scale and not considered in the fit). (**b**) The flux increases linearly with the GDP of the region of destination (continuous line, linear fit; see Equations (6) and (7)).

We hypothesise that strong correlations among regions also arise for an infective component, continually spreading from region to region at small time scales (less than a month), driven by the movement of people from one region to another. First, we examined the data about the flux of daily commuters between regions; such data show a clear dependence both on spatial distance (exponential decay; see Figure 3a; the continuous line is the result of a fit, see Equations (4) and (5)) and the GDP of the region of arrival, having the role of an ‘attractiveness’ factor (linear dependence; see Figure 3b, where the continuous line is the result of a fit; see Equations (6) and (7)).

**Figure 4:**
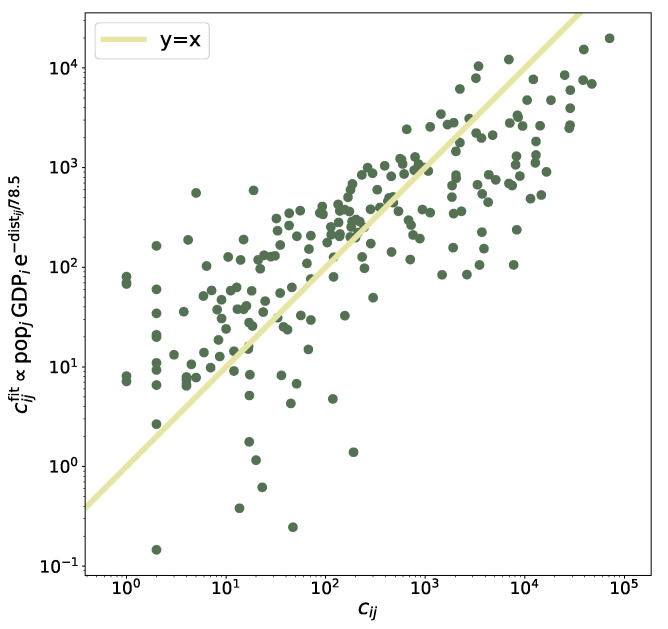
Actual commuters flux *vs*. the flux reconstructed by a fitted model that decays exponentially with the distance and grows linearly with the GDP of the region of destination (see Equation (8)). The continuous line is the identity line. Only non-zero entries of the commuters’ matrix are displayed and considered in the fitting procedure.

The two determinants (distance and GDP) are considered together in Figure 4, where the actual number of commuters (from one region to another; the flux is not symmetric) is compared to the result of the fitted model (see Equation (8)); the good agreement of the reconstructed flux with the real one (the continuous line is the identity line) supports the assumptions of the model.

Starting from these results, we make the hypothesis that the total flux of people between regions is made of the commuters flux plus an ‘episodic’ flux, unknown but with the same functional form (exponential decay with distance; linear dependence on GDP). Then, we built, on the total-flux matrix, a SIR-like model [10] that takes into account the exchange of infected people between regions. Temperature impacts the model in two ways. The first increases with the temperature and is akin to the high-temperature (rightmost) arm of the model of Figure 2a. The other modulates the contagiousness of the disease (higher for lower temperatures).

In the model, each month a new disease starts spreading from a given region (chosen according to a probability distribution); at the end of the month, a fraction of the ‘recovered’ people dies. Added to this effect are the high-temperature mortality and, finally, a generic, temperature-independent, region-specific mortality.

**Figure 5:**
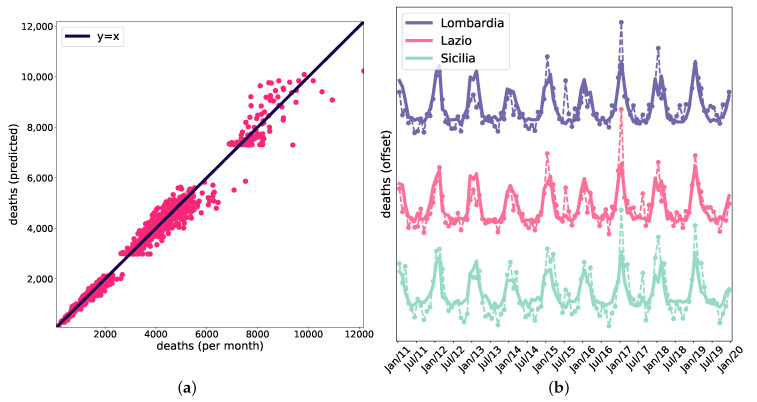
(**a**) Actual deaths vs. the deaths expected by the model (corr = 0.993; the continuous line is the identity). (**b**) Time-series of the deaths for three regions; dashed lines: data; continuous lines: model.

We fitted the model’s parameters (see Material and Methods) to the data. Figure 5a shows the death counts for all the regions and all the considered months against the death counts generated by the model. The model can reproduce a large part of the observed variability (corr = 0.993; the continuous line is the identity line). This can be appreciated, as the deaths evolve over time, in Figure 5b, for three different regions (dashed lines: data; continuous line: model). Note that the three time series are offset vertically to make the comparison data vs. model clearer.

**Figure 6:**
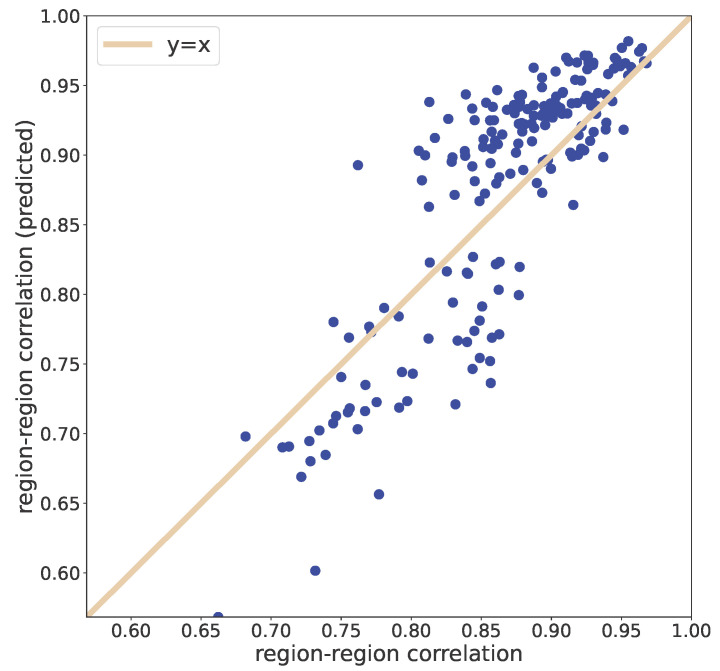
Between-region correlation: data vs. reconstructed from the model (corr = 0.841; the continuous line is the identity line).

Finally, we compare, in Figure 6, the observed between-region correlations and the correlations between the time series produced by the model for each region. To a large extent, the model can capture the variability of the correlation among the regions (corr = 0.841; the continuous line is the identity line).

## 4. Discussion

We aptly reconstructed the strong correlation among temporal series of all-cause monthly death rates relative to the 20 Italian regions by a model encompassing non-infectious (mainly summer) and infectious (winter) components. This last component was modelled in terms of a set of SIR equations taking into account both the across-regions commuter exchange (daily flow) and the more irregular traveller flux (longer time flow). The different mathematical treatments of summer and winter components allowed for a neat increase in the reconstruction of both global death rates and among-regionscorrelation strength concerning crude seasonality.

The reconstruction of the observed correlation network by our model (Figure 6), excluding the hypothesis of contemporary arising ‘epidemic sources’ across all the regions (that have near-zero probability), confirms the reliability of the proposed model. Overall, we can consider Italy as a proper ‘integrated system’ that, thanks to both a rich exchange flux among regions and the sharing of ‘heat waves’, reaches a general coherence in death rate fluctuations. This coherent behaviour acts as a largely invariant ‘mean field’ governing all-cause monthly (and thus unaffected by longer time fluctuations in age class distribution) death rate fluctuations.

The existence of a very stable correlation network among Italian regions can be profitably used as a tool for the epidemiological surveillance of the territory: the arising of an anomalous value of the correlation degree of a region can be intended as the presence of an emerging source of risk (of both infectious and/or environmental origin). Thanks to the intrinsic redundancy of the correlation matrix, any (even transient) change reverberate on the entire network, allowing for a more sensible detection of instabilities: one clear example is the case of Recurrence Quantification Analysis (RQA, [12]). RQA relies upon the construction of a distance (correlation in the case of angular metrics) matrix between subsequent epochs of a time (or space [13])-dependent signal. When in the presence of regime changes driven by a slowly varying control parameter, RQA metrics (at odds with usual statistical indexes) exactly determine the entity and time (spatial) location of regime change [14,15]. Analogous considerations hold for correlation matrices and any other network system [16] induced by external (e.g., epidemics) or internal (e.g., deterioration of living conditions) driving forces [17].

The application of statistical-mechanics-inspired tools in public health is still in its infancy [18,19] and/or confined to very specific issues [20,21]. In this work, relying upon a very general consideration by Alexander Gorban and colleagues, ‘It is useful to analyse correlation graphs’ [22], we demonstrate how a raw (albeit very reliable) indicator, all-cause mortality, is amenable to a statistical mechanics approach opening new avenues to epidemiological and environmental research.

## 5. Conclusions

All-cause mortality is considered a general indicator of the general health status of a population in the context of a particular age structure. This is why many epidemiological studies investigate both temporal and spatial fluctuation of all-cause mortality, looking for a correlation with socioeconomic [23], environmental [24,25,26] and physiological/pathological conditions [27].

Moreover, the reliability of all-cause mortality statistics, due to its coarse-grain character, makes the analysis of its fluctuations a very important viewpoint to estimate the impact of epidemic threats [28].

Our approach stems from the above considerations on all-cause mortality to explore a still neglected dimension of this index: the relative weight of purely stochastic and deterministic (coherent) components of spatio-temporal fluctuations of all-cause mortality. The presence of external drivers increasing the correlation of such fluctuations was already observed [24] in terms of departure from ‘optimal temperature’, here we highlight another correlation source linked to the population fluxes among different areas modelled in terms of both daily commuters and ‘relative attractiveness’ of different regions in terms of GDP.

This choice allowed us to get rid of a very dense correlation network among the 20 Italian regions for monthly-based fluctuation rates in the 2011–2019 period. The presence of very high temporal correlations among different regions was partly unexpected and constituted a very important result for monitoring the onset of emerging public health threats in terms of alterations of such correlation structures.

## Data Availability

The daily deaths for each Italian district (‘comune’), starting from 2011, can be downloaded from the site of the Italian Institute of Statistics (ISTAT), following this link: https://www.istat.it/storage/dati_mortalita/Dataset-decessi-comunali-giornalieri_regioni%28excel%29_5-21-ottobre-2021.zip (accessed on 24 June 2022). The geographical coordinates for each *comune* can be found in the following GitHub project: https://github.com/MatteoHenryChinaski/Comuni-Italiani-2018-Sql-Json-excel, notably the file italy_geo.xlsx (accessed on 24 June 2022). The data concerning the temperature have been obtained, on 23 June 2022, from the National Centers for Environmental Information (https://www.noaa.gov/), with the following order specifications: Begin date: 2012-01-01 00:00; End date: 2021-12-31 23:59; Data types: PRCP, SNWD, TAVG, TMAX, TMIN; Custom Flags: Station Name, Geographic Location, Include Data Flags. The number of daily commuters between regions has been obtained from the data made available by ISTAT at the link: https://www.istat.it/storage/cartografia/matrici_pendolarismo/matrici_pendolarismo_2011.zip (data description can be found at https://www.istat.it/it/archivio/157423; accessed on 28 June 2022). The number of commuters we used is the sum of commuters from any *comune* belonging to one region towards any *comune* of another region. The GDP for each region has been retrieved from the Wikipedia page https://it.wikipedia.org/wiki/Regioni_d%27Italia (GDP is ‘Prodotto interno lordo’ or PIL, in Italian; accessed on 10 October 2022).
